# Mosquito surveillance of West Nile and Usutu viruses in four territorial units of Slovakia and description of a confirmed autochthonous human case of West Nile fever, 2018 to 2019

**DOI:** 10.2807/1560-7917.ES.2021.26.19.2000063

**Published:** 2021-05-13

**Authors:** Viktória Čabanová, Elena Tichá, Richard Stewart Bradbury, Dana Zubriková, Daniela Valentová, Gabriela Chovancová, Ľubomíra Grešáková, Bronislava Víchová, Silvie Šikutová, Tomáš Csank, Zuzana Hurníková, Martina Miterpáková, Ivo Rudolf

**Affiliations:** 1 Institute of Virology, Biomedical Research Center, Slovak Academy of Sciences, Bratislava, Slovakia; 2 Institute of Parasitology, Slovak Academy of Sciences, Košice, Slovakia; 3 The National Reference Centre for Arboviruses and Haemorrhagic Fevers of the Public Health Authority of the Slovak Republic, Bratislava, Slovakia; 4 School of Health Sciences, Federation University, Berwick, Australia; 5 Veterinary and Food Institute in Bratislava, Bratislava, Slovakia; 6 Research Station and Museum of TANAP, Tatranská Lomnica, Slovakia; 7 Institute of Animal Physiology, Centre of Biosciences, Slovak Academy of Sciences, Košice, Slovakia; 8 The Czech Academy of Sciences, Institute of Vertebrate Biology, Brno, Czech Republic; 9 University of Veterinary Medicine and Pharmacy, Košice, Slovakia

**Keywords:** West Nile, Usutu, arbovirus, flavivirus, mosquitoes, Slovakia, Europe

## Abstract

**Background:**

Despite the known circulation of West Nile virus (WNV) and Usutu virus (USUV) in Slovakia, no formal entomological surveillance programme has been established there thus far.

**Aim:**

To conduct contemporaneous surveillance of WNV and USUV in different areas of Slovakia and to assess the geographical spread of these viruses through mosquito vectors. The first autochthonous human WNV infection in the country is also described.

**Methods:**

Mosquitoes were trapped in four Slovak territorial units in 2018 and 2019. Species were characterised morphologically and mosquito pools screened for WNV and USUV by real-time reverse-transcription PCRs. In pools with any of the two viruses detected, presence of *pipiens* complex group mosquitoes was verified using molecular approaches.

**Results:**

Altogether, 421 pools containing in total 4,508 mosquitoes were screened. Three pools tested positive for WNV and 16 for USUV. USUV was more prevalent than WNV, with a broader spectrum of vectors and was detected over a longer period (June–October vs August for WNV). The main vectors of both viruses were *Culex pipiens* sensu lato. Importantly, WNV and USUV were identified in a highly urbanised area of Bratislava city, Slovakias’ capital city. Moreover, in early September 2019, a patient, who had been bitten by mosquitoes in south-western Slovakia and who had not travelled abroad, was laboratory-confirmed with WNV infection.

**Conclusion:**

The entomological survey results and case report increase current understanding of the WNV and USUV situation in Slovakia. They underline the importance of vector surveillance to assess public health risks posed by these viruses.

## Introduction

West Nile virus (WNV) and Usutu virus (USUV) are members of the *Flaviviridae* family of viruses and are phylogenetically and antigenically related to the Japanese encephalitis virus complex. WNV and USUV share an enzootic transmission cycle involving amplification in avian reservoir hosts and mosquitoes as vectors. In Europe, the major vectors belong to the *Culex pipiens* complex. Mosquitoes can transmit both WNV and USUV to horses, humans, or other mammals. These are, however, considered dead-end hosts as the viraemia in such hosts does not reach a level sufficient to further infect vectors during a blood meal [1-4]. For WNV, *Cx. modestus* is considered as the principal bridge vector between birds and humans.

Recently, the scientific and public awareness of WNV and USUV has increased in Europe due to their emergent nature. In 2009, the first human neurological disorder caused by USUV in Europe changed the previously held perspective that this virus is primarily a bird pathogen [4,5]. For WNV, the number of human WNV cases in the European Union rose substantially in 2018 compared with previous years [6].

In Slovakia, the presence of WNV and WNV antibodies in dead-end (horses, sheep and human) and reservoir hosts (birds) has been investigated several times since 1967, mainly in the southern regions [7-10]. Nevertheless, until 2018, flavivirus screening of mosquitoes had only been conducted once, in 1972, in the country’s western regions. In the 1972 study, WNV had been detected in a pool of *Aedes cantans* [11]. The geographical spread of USUV in Slovakia is at present not well understood. Csank et al. [12,13] detected USUV antibodies in birds from the Levice district (South Central (SC) Slovakia) between 2012 and 2014 and in lizards from the Slovak Karst National Park (South East (SE) Slovakia) between 2017 and 2018 [14]. In 2018, an entomological surveillance study conducted in the southern Slovak district of Komárno, revealed a remarkably high prevalence of WNV in mosquitoes with a minimum infection rate (MIR) of 0.46, as well as the first identification of USUV-infected mosquitoes in the country (MIR: 0.25). Furthermore, this work demonstrated the co-existence of both viruses in the same environment [15]. To our knowledge, no human case of USUV has been diagnosed in Slovakia so far.

In the current study, a contemporaneous nationwide entomological survey for WNV and USUV is reported as well as its results. In addition, the first autochthonous human West Nile fever (WNF) case in Slovakia, who was diagnosed in the summer of 2019 is described.

## Methods

### Usutu and West Nile virus vector surveillance

#### Geographical locations and period of mosquito sampling

**Figure 1:**
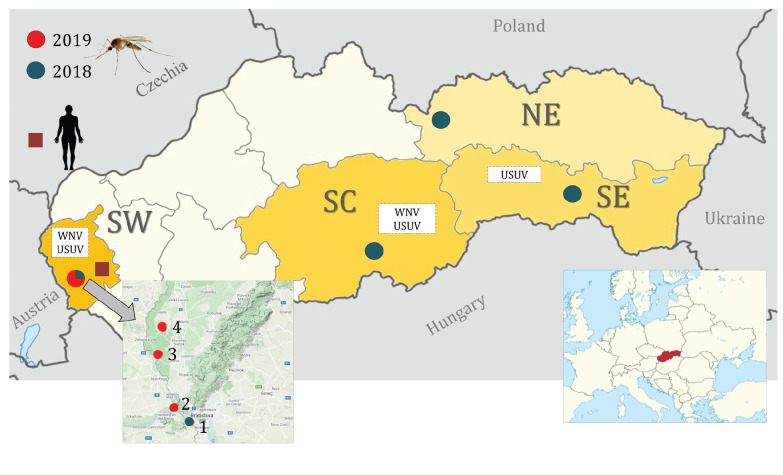
West Nile virus and Usutu virus entomological surveillance areas and location of the occurrence of a human case of West Nile fever, Slovakia, 2018, 2019

NE: North Eastern Slovakia; SC: South Central Slovakia; SE: South Eastern Slovakia; SW: South Western Slovakia; USUV: Usutu virus; WNV: West Nile virus.

A nationwide WNV vector surveillance was established in four separate territorial units of the country including South Western (SW), SC, SE and North Eastern (NE) Slovakia (Figure 1).

The mosquito sampling sites are marked by circles (1 – Bratislava city, 2 – Devín Castle, 3 – Jakubov, 4 – Vysoká pri Morave), while the human West Nile fever case infection site is indicated by a square.

Within SW Slovakia, entomological surveillance was conducted at four locations. The first location, was in an urban setting (Bratislava city) between May and November 2018. Mosquitoes were sampled using one BG-Mosquitaire CO_2_ (Biogents, Regensburg, Germany) trap placed close to the city centre, in a sport and walking area located on the Danube river bank. The Danube and the Karloveské rameno river branch off ca 100 m from this sampling site (48.1462N, 17.07062E). Between 12 and 14 August 2019, three further locations were investigated. For this part of the study, six Encephalitis Vector Survey (EVS) traps with dry ice (BioQuip Products, Inc., Rancho Dominguez, California, United States (US)) were set up. One of the sites was the Záhorská lowland area near Devín Castle, in a Bratislava city suburb (48.17514N, 16.97695E), where intensive mosquito sampling was conducted in fishponds and wetland environments. Another sampling locality was situated in a reed bed ecosystem in the vicinity of Vysoká pri Morave village (48.30541N, 16.9628 E). The last location was in a rural environment in a man-made fishpond in Jakubov village (48.41029N, 16.9165E).

In SC Slovakia, sampling was conducted in a rural environment between May and October 2018. For this, a BG-Mosquitaire CO_2_ (Biogents) trap was placed in a family house garden in Podrečany village (48.403614N, 19.605153E).

Concerning SE Slovakia, mosquitoes were captured along the Hornád river from June to October 2018 with one BG-Sentinel CO_2_ (Biogents) trap. The sampling site was located in Košice city, close to the Anička city park (48.744750N, 21.257472E).

The NE Slovakia mosquito collection took place in the mountainous environment of the Tatras National Park (TANAP). A BG-Mosquitaire CO_2_ trap was set up between June and November 2018 in the TANAP Research Station and Museum in a rural habitat of Tatranská Lomnica (49.166646N, 20.284605E). This site was located at 842 metres above sea level.

#### Vector species morphological identification and preparation of mosquito pools

Trapped mosquitoes were stored at − 80 °C before further processing. Female mosquitoes were morphologically distinguished using the identification keys from Becker et al. [16] and pooled according to species, sampling site, date and locality with a maximum of 50 individuals per pool.

#### Testing mosquito pools for viruses

In preparation for nucleic acid extraction, pools were homogenised in sterile phosphate buffered saline (PBS; pH = 7.2; 200 to 800 µL depending on the number of mosquitoes in a pool) using a Qiagen Tissue Lyser II (Qiagen, Hilden, Germany) with 5 mm stainless beads at 30 Hz/min. RNA was extracted from 140 µL of the supernatant using the GeneJet Genomic RNA Purification Kit (ThermoFisher, Dreieich, Germany), according to the manufacturer’s instructions.

The presence of WNV and USUV nucleic acids was tested by two separate (one for WNV and one for USUV) one-step real-time reverse-transcription (RT)-PCR protocols (respectively described below). The PCRs were carried out on the Bio-Rad CFX96 Real-time system (Bio-Rad, Hercules, California, US) with a qScript XLT One-Step RT-qPCR ToughMix, ROX (Quantabio, Beverly, Massachusetts, US).

For WNV screening, a primer set (forward primer: WNV-8F; reverse: WNV-118R) targeting the 5´ non-coding region (5´NCR) and a TaqMan probe (WNV-67T) designed by Kolodziejek et al. [17] were used at the following final concentrations: WNV-8F 200 nM, WNV-118R 600 nM and WNV-67T 250 nM. Two positive samples (WNV lineage 2, strain J180721 RNA diluted 1:100,000) and two negative samples (nuclease-free water) were included as positive and negative controls with each run without modification of a prior-published thermal profile [18].

For the USUV real-time RT-PCR, primers targeting the non-structural protein 5 (NS5) region, USU-9721F, USU-9795R and TaqMan probe USU-9746 (final concentration 200 nM of each) were used without modification of the thermal profile [18]. Two samples of nuclease free water and USUV Europe 2, strain USUV220/2018/SK RNA (dilution 1:1,000) were included with each run as negative and positive controls. USUV and WNV real-time RT-PCRs with a cycle threshold (Ct) values over 40 were considered negative.

#### Minimum infection rate estimation

The minimum infection rate (MIR) in an area, assuming a single positive mosquito in a pooled sample, was calculated by extrapolation from the real-time PCR results (the total number of positive pools in the area/total number of mosquitoes sampled in this area × 1,000).

#### Selective viral gene amplification and sequencing

To obtain sequence information for subsequent determination of USUV or WNV lineages positive samples were further tested by specific RT-PCRs targeting the WNV envelope (ENV) gene (position: 1,531–1,836) [19] and USUV NS5 protein (position: 9,177–9,689) [18], using a one-step RT-PCR kit (Qiagen). The amplicons were visualised on 1.5% agarose gel followed by Sanger sequencing in a commercial laboratory (Eurofins Genomics, Ebersberg, Germany).

#### Analysis of viral genetic sequences

All sequences obtained were verified using Basic Local Alignment Search Tool (BLAST). Phylogenetic analyses were conducted using a maximum likelihood (ML) algorithm Kimura-2 model (Molecular Evolutionary Genetics Analysis; MEGA 6.0 [20]) with bootstrap resampling of 1,000 replicates.

#### Confirmation of mosquito species by molecular methods

Furthermore, for positive real-time RT-PCR WNV and USUV pools, a molecular identification of the acetylcholinesterase 2 (ACE-2) and CQ11 loci of the *pipiens* complex group was performed, extensively described elsewhere [21-23].

### West Nile virus infection clinical diagnostics 

Diagnostics for WNV were conducted at the National Reference Centre for Arboviruses and Haemorrhagic Fevers of the Public Health Authority of the Slovak Republic. In the acute phase of infection, patient serum was tested using both an IgM Anti-West Nile Virus ELISA and IgG Anti-West Nile Virus ELISA (Euroimmun, Lübeck, Germany). Whole blood, urine and a second serum sample, all obtained 5 days after the first sample, were also tested. The second serum sample was assayed for IgG antibody avidity against WNV (Euroimmun). The whole blood sample, urine as well as both serum samples were subjected to RNA extraction with the QIAamp Viral RNA Mini (Qiagen) and tested for WNV by one step real-time RT-PCR using the RealStar WNV RT-PCR kit (Altona Diagnostics, Hamburg, Germany) in an iQ5 Biorad Real-Time PCR detection system (Bio-Rad), following the manufacturer’s instructions.

The RT-PCR amplicon was sequenced at the World Health Organization Collaborating Centre for Arbovirus and Haemorrhagic Fever Reference and Research, Bernhard Nocht Institute for Tropical Medicine in Hamburg, Germany.

### Ethical statement

The human samples were tested in the National Reference Centre for Arboviruses and Haemorrhagic Fevers of the Public Health Authority of the Slovak Republic established by decision of the Ministry of Health of the Slovak Republic in accordance with § 8 of the Act Number 126/2006 Coll. of Laws on public health system, effective since 1 May 2007 (International Organization for Standardization (ISO) 15189:2012). No patient identifiers were included in the study so ethical approval was not necessary.

## Results

### Mosquitoes sampled

Altogether, 421 separate pools including in total 4,508 individual female mosquitoes were trapped in 2018 and 2019 (Table). The following species were detected: *Aedes vexans* (one pool), *Anopheles hyrcanus* (seven pools), *An. maculipennis* sensu lato (s.l.) (34 pools), *An. plumbeus* (19 pools*), Cx. modestus* (30 pools), *Cx. pipiens* s.l. (317 pools), *Culiseta annulata* (four pools), *Cs. longiareolata* (eight pools) and *Coquillettidia richiardii* (one pool). All obtained mosquito pools were processed for flavivirus screening.

**Table:** Adult female mosquito pools screened for West Nile and Usutu viruses, presented according to the territorial units in which they were collected, Slovakia, 2018 and 2019 (n = 421 pools)

Mosquito sampling territorial unit, area (year)	Mosquito species	Number of tested individuals	Number of tested pools	Number of pools positive for WNV by real-time RT-PCR^a^/RT-PCR^b^	Number of pools positive for USUV by real-time RT-PCR^a^/RT-PCR^b^
**South Western Slovakia**	**All species**	**2,766**	**246**	**2/2 (MIR** ^c^: **0.72)**	**14/10 (MIR** ^c^: **5.06)**
Bratislava city (2018)	*An. maculipennis* s.l.	44	25	0	1
	*An. plumbeus*	12	11	0	0
	*Cq. richiardii*	1	1	0	0
	*Cx. modestus*	9	8	0	0
	*Cx. pipiens* s.l.	1,830	134	2	9
	*Cs. annulata*	1	1	0	0
	*Cs. longiareolata*	8	7	0	0
	Subtotal	1,905	187	2/2 (MIR^c^: 1.05)	10/9 (MIR^c^: 5.25)
Devín Castle (2019)	*Ae. vexans*	2	1	0	0
	*An. hyrcanus*	9	4	0	0
	*An. maculipennis* s.l.	3	2	0	0
	*Cx. modestus*	178	9	0	0
	*Cx. pipiens* s.l.	59	9	0	0
	Subtotal	251	25	0/0 (MIR^c^: NA)	0/0 (MIR^c^: NA)
Jakubov (2019)	*An. maculipennis* s.l.	2	2	0	0
	*Cx. modestus*	110	6	0	0
	*Cx. pipiens* s.l.	138	6	0	0
	Subtotal	250	14	0/0 (MIR^c^: NA)	0/0 (MIR^c^: NA)
Vysoká pri Morave (2019)	*An. hyrcanus*	30	3	0	1
	*An. maculipennis* s.l.	29	4	0	0
	*Cx. modestus*	208	7	0	2
	*Cx. pipiens* s.l.	93	6	0	1
	Subtotal	360	20	0/0 (MIR^c^: NA)	4/1 (MIR^c^: 11.11)
**South Central Slovakia**	**All species**	**323**	**53**	**1/1 (MIR** ^c^: **3.10)**	**1/1(MIR** ^c^: **3.10)**
Podrečany village (2018)	*An. plumbeus*	9	8	0	0
	*Cx. pipiens* s.l.	314	45	1	1
**South Eastern Slovakia**	**All species**	**1,188**	**81**	**0/0 (MIR^c^: NA)**	**1/1 (MIR** ^c^: **0.84)**
Košice city (2018)	*An. maculipennis* s.l.	1	1	0	0
	*Cx. pipiens* s.l.	1,186	79	0	1
	*Cs. longiareolata*	1	1	0	0
**North Eastern Slovakia**	All species	**231**	**41**	**0/0** **(MIR** ^c^: **NA)**	**0/0 (MIR** ^c^: **NA)**
Tatranská Lomnica (2018)	*Cx. pipiens* s.l.	228	38	0	0
	*Cs. annulata*	3	3	0	0
**Total**		**4,508**	**421**	**3/3 (MIR** ^c^: **0.67)**	**16/12 (MIR** ^c^: **3.55)**

*Ae*.: *Aedes*; *An*.: *Anopheles*; *Cq*.: *Coquillettidia*; *Cs*.: *Culiseta*; *Cx*.: *Culex*; MIR: minimum infection rate; NA: not applicable; s.l.: sensu lato; RT-PCR: reverse-transcription PCR, USUV: Usutu virus, WNV: West Nile virus.

^a^ The real-time RT-PCR for WNV targeted the 5´ non-coding region of the virus while the real-time RT-PCR for USUV targeted the non-structural protein 5. PCRs with a cycle threshold value above 40 were considered as a negative result.

^b^ Mosquito pools, which were positive for WNV or USUV by real-time RT-PCR, were further subjected to specific RT-PCRs targeting the envelope gene (for WNV) or the non-structural protein 5 gene (for USUV).

^c^ The MIR was calculated based on the real-time RT-PCR results.

### West Nile virus infected mosquitoes

WNV real-time RT-PCR revealed three positive pools among the 421 pools tested (MIR: 0.67), with Ct values 21.21, 25.12 and 30.91 (Table). All three pools were subsequently confirmed by RT-PCR and sequencing.

Two of the positive pools originated from Bratislava city in the SW Slovak territorial unit. The MIR in that territorial unit was estimated at 0.72. The last positive pool came from Podrečany village in SC Slovakia, yielding a MIR of 3.10 for the SC Slovakia territorial unit. No WNV RNA was detected in SE and NE territorial units of Slovakia.

All WNV-infected mosquitoes were trapped between 10 and 25 August 2018.

According to the morphological identification, the positive pools all consisted of *Cx. pipiens* s.l., with molecular identification revealing two pure pools of *Cx. pipiens pipiens* bioform and a mixed signal of *Cx. pipiens pipiens*/*Cx torrentium* in one of the pools from Bratislava.

**Figure 2:**
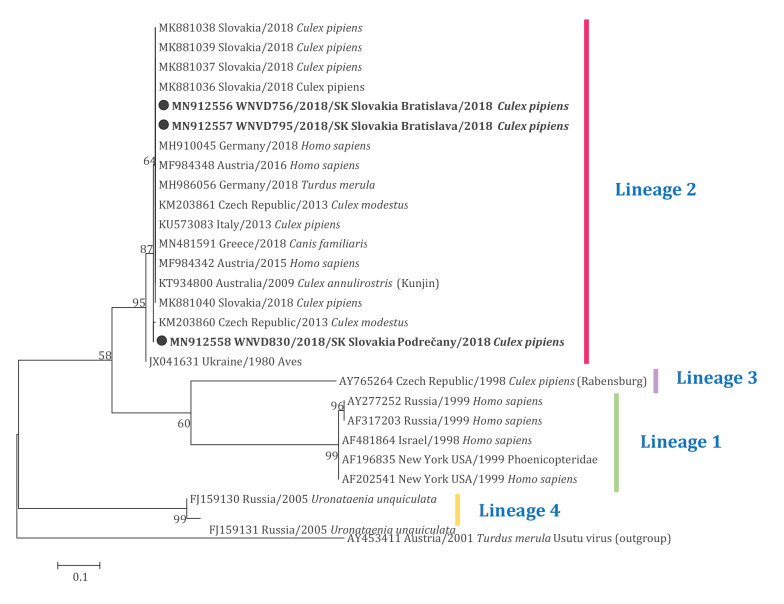
Maximum likelihood phylogenetic tree based on West Nile virus envelope gene sequences, showing the genetic relatedness of viruses infecting mosquitoes in Slovakia to West Nile virus lineage 2 strains, 2018 (n = 27 sequences in tree)

BLAST analyses of WNV sequences extracted from mosquitoes yielded 99.65%–100.00% similarity to sequences of WNV lineage 2 originating from different European countries, such as Germany (GenBank accession number: MH910045) and Austria (GenBank accession number: MF984342). Phylogenetic analyses placed all WNV strains from Slovak mosquitoes within the main lineage 2 cluster, with weak clustering of Podrečany village and a Czech strain (GenBank accession number: KM203860) (Figure 2). The Slovak WNV strain sequences are available in GenBank under accession numbers MN912556–MN912558.

The tree was generated using the Kimura-2 model with bootstrap resampling of 1,000 replicates. The tree is drawn to scale, with branch lengths corresponding to the number of substitutions per site. Bootstrap values are shown at the nodes. GenBank accession numbers of the sequences in the tree are provided and the sequences retrieved in the current study are in bold font and further indicated by black circles.

### Usutu virus infected mosquitoes

USUV screening of the 421 mosquito pools retrieved in the study by real-time RT-PCR led to detect USUV RNA in 16 pools with an overall MIR of 3.55. Ct values ranged between 19.17 and 38.39. The contemporaneous RT-PCR detected RNA only in 12 of these pools (Table). USUV was found circulating in all investigated locations of southern Slovakia. The virus was not detected in the sampling site in NE Slovakia, which was at higher altitude in the mountain habitat of the Tatras National Park.

Overall, the majority of infected mosquitoes were caught in SW Slovakia (14 pools; MIR: 5.06). Ten of the infected pools came from an urban area of Bratislava city (year 2018) and another four from a sampling site situated in a reed bed ecosystem close to Vysoká pri Morave village (year 2019).

In SC Slovakia, a single positive pool was detected in Podrečany village, yielding a MIR of 3.10 for the SC Slovakia territorial unit (year 2018).

One positive pool was found also in the urban habitat of Košice city in SE Slovakia (year 2018). The MIR in the SE Slovakia territorial unit was estimated to be 0.84.

USUV infected mosquitoes were trapped between 8 June and 4 October 2018 and 12 to 14 August 2019, with the USUV prevalence peak in August (eight pools August 2018, four pools August 2019).

The vector composition was more variable than observed for the WNV positive pools. Twelve pools of *Cx. pipiens* s.l. were found to be infected with USUV. Molecular identification revealed pure biotype *Cx. pipiens pipiens* in seven pools. A mixed signal of *Cx. pipiens pipiens*/*Cx. torrentium* was found in five pools and consistent with the entomological findings of the WNV surveillance, all of these pools came once more from Bratislava. In SW Slovakia, USUV was identified in other mosquito species, namely *An. maculipennis* s.l. (one pool), *An. hyrcanus* (one pool) and *Cx. modestus* (two pools).

**Figure 3:**
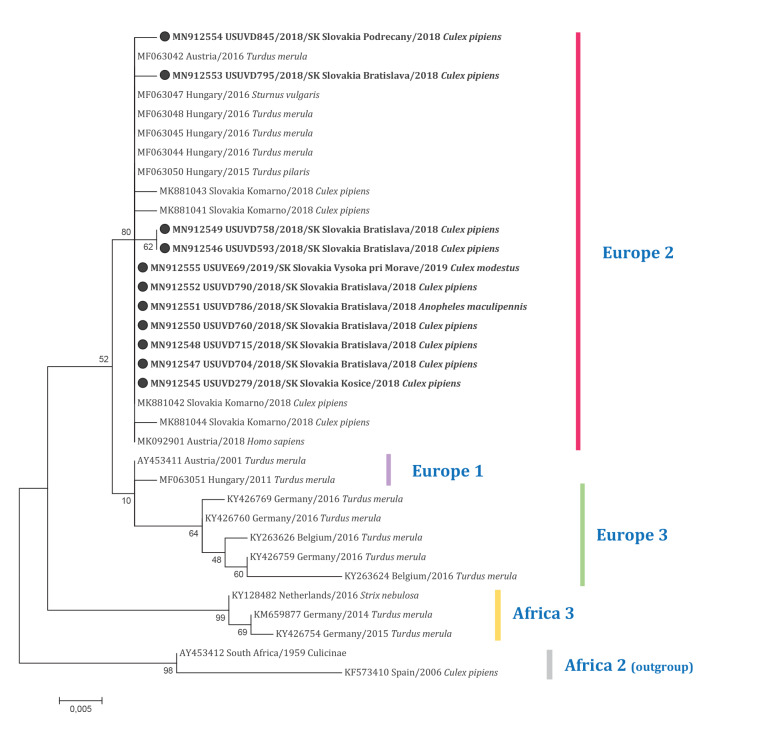
Maximum likelihood phylogenetic tree based on the Usutu virus non-structural protein 5 gene sequences, showing the relatedness of strains infecting mosquitoes in Slovakia to Usutu virus lineage Europe 2, 2018, 2019 (n = 34 sequences in tree)

BLAST alignment of the USUV sequences obtained from mosquitoes yielded highest similarity (99.78–100.00%) with USUV sequences of strains isolated from a fieldfare (*Turdus pilaris*) in 2015 from Hungary (GenBank accession number: MF063050) and from a blackbird (*Turdus merula*) in 2016 from Austria (GenBank accession number: MF063042). From ML phylogenetic analysis, the strains retrieved in this study appeared to belong to the USUV lineage Europe 2 (Figure 3). Moreover, weak clustering of some Slovak sequences was observed within the main cluster of Europe 2. The sequences obtained in this investigation were submitted to the GenBank under accession numbers MN912545–MN91254.

The tree was generated the using Kimura-2 model with bootstrap resampling of 1,000 replicates. The tree is drawn to scale, with branch lengths corresponding to the number of substitutions per site. Bootstrap values are shown at the nodes. GenBank accession numbers of the sequences in the tree are provided and the sequences retrieved in the current study are in bold font and further indicated by black circles.

### Human case of West Nile fever in Slovakia

At the end of August 2019, a patient presented to hospital with fever (40 °C) accompanied by headache, weakness, myalgia, distractibility and arthralgia. A papular exanthema had developed on their thorax. The patient was admitted to hospital and a subsequent neurological examination (lumbar puncture, magnetic resonance imagining) showed normal findings. Laboratory testing showed full blood count parameters within the normal range. Serological analyses for rubella virus, influenza viruses, parainfluenza viruses, *Borelia *spp*.*, tick-borne encephalitis virus, *Chlamydophila pneumoniae*, *Mycoplasma pneumoniae, Leptospira* spp., hepatitis viruses and human immunodeficiency virus were all negative.

Four days prior, the patient had been bitten several times by mosquitoes, during a visit to a village in the Bratislava region in SW Slovakia (Figure 1). The patient reported not travelling outside Slovakia in the 14 days before symptom onset.

A consultation with an infectious disease specialist raised the suspicion of WNF and laboratory testing for WNV was requested. A first blood serum collection taken on the fourth day of hospitalisation was tested by WNV ELISA and was positive for WNV IgM but not WNV IgG. The second serum sample taken 1 week later was positive for WNV IgM and IgG, and had a low avidity for WNV IgG. The whole blood and urine samples collected at the same time, as well as both serum samples, were tested by a one-step WNV real-time RT-PCR with a positive result only from whole blood. The RT-PCR amplicon was sequenced and the sequence was found to belong to WNV lineage 2 (data not shown).

The limited remaining patient sample material did not allow sequencing of the WNV ENV protein gene for inclusion in the phylogenetic tree. Five days after hospitalisation, the patient began to recover and was released from hospital.

The patient represents the first autochthonous laboratory-confirmed case with clinical criteria of WNV infection from Slovakia (according the European Union case definition [24]).

The case was reported to The European Surveillance System (TESSy), Rapid Alert Blood (RAB) and other European public health surveillance databases. The National Transfusion Service was immediately alerted and the Public Health Authority of the Slovak Republic published a guideline to raise medical practitioners’ awareness in mid-September 2019.

## Discussion

Ecological factors, vector and host composition in a natural environment are responsible for the clustered distribution of arboviruses. Targeted vector surveillance can reveal foci, seasonality and ecological niches of such viruses [25,26]. The information from the surveillance can assist in identifying areas and periods of high viral circulation in vectors and allow to preempt outbreaks [25,26]. Taking this into consideration, nationwide mosquito surveillance is of upmost importance for arbovirus control programmes.

While an emerging risk of human WNV infection in Slovakia has already been suggested since some time by prior investigations, no formal entomological surveillance programme is so far established in the country. In previous studies, WNV was detected in reservoir species as well as WNV antibodies in dead-end hosts [7-10], and the virus was also shown to circulate in neighbouring countries, particularly Hungary [27-29] and Austria [30,31], where human cases of WNV infection were confirmed.

In Hungary, an outbreak of WNV in birds occurred in 2003, followed by the first autochthonous human cases. Subsequently, the circulation of two WNV lineages (lineage 1 and 2) was recognised there [27]. Between 2003 and 2017, an average of 15 to 20 human cases of WNF and West Nile neuroinvasive disease (WNND) was diagnosed annually [28,29], and an extraordinary increase in human WNF cases was reported in 2018. During this year, the number of autochthonous (n = 215) and imported (n = 10) cases of WNV infection was nine times higher than in 2017 (n = 23), moreover, their cumulative number (n = 225) exceeded that of the total number of cases in the previous 14 years (n = 213) [28]. Hungary borders Slovakia to the south and both our data and previous records show that the majority of WNV infections in birds, horses and mosquitoes in Slovakia occurs along this southern border [32]. In the current study, we only detected WNV in a rural village (Podrečany) of SC Slovakia. 

Austria borders Slovakia to the west and south-west. In Austria, WNV lineage 2 was first detected in birds and mosquitoes in 2008 and the first human WNF cases were diagnosed in 2006 and 2010 [30]. A total of 23 autochthonous human cases were recorded between 2008 and 2017. The number of recorded human cases of WNV infection in 2018 was 21 [31], demonstrating a similar fold increase as the one observed in Hungary that year. Our study identified WNV in mosquitoes in SW Slovakia. Of particular concern, was the finding of infected vectors in the highly urbanised centre of Bratislava city. Moreover, the first WNF human case recorded in Slovakia, occurred in the south-west part of the country, ca 20 to 25 km from both Hungary and Austria. This Slovak patient’s clinical symptoms were typical of WNF infection. Clinical and laboratory diagnostic testing was appropriate and rapid, with the initial serological detection followed up by molecular confirmation. As the human cases in Hungary and Austria, the virus strain detected from the first autochthonous Slovak case was of lineage 2, similar to the Slovak mosquitoes.

Initially, the risk of USUV infections was not expected to be high in Slovakia due to the low number of records in the literature [12-14]. However, our results showed that USUV was considerably more prevalent in mosquito vectors (MIR: 3.55) than WNV (MIR: 0.67) and the spectrum of mosquito species infected with USUV was broader than for WNV. USUV was present in all three territorial units of southern Slovakia. USUV human cases are known to have occurred in surrounding countries [33]. In Hungary, the first human symptomatic case of USUV (aseptic meningitis) was diagnosed in 2018. The strain was identified as USUV lineage Europe 2 [28]. In Austria, 18 human USUV cases were recorded in the year 2018 among blood donors, with one donor co-infected with both WNV and USUV. The majority of USUV cases were asymptomatic. The USUV strains from the Austrian cases were related to the Europe 2 and Africa 3 lineages [31]. All USUV strains in Slovak mosquitoes from our study were related to lineage Europe 2. Nonetheless, circulation of more USUV lineages has been confirmed in other countries bordering Slovakia, such as Czechia [33].

As expected given the high altitude, no occurrence of WNV and USUV was detected in the mountainous site of High Tatras National Park. On the other hand, at some of the other study locations, co-circulation of WNV and USUV viruses was observed, such as in Bratislava city (SW Slovakia) and Podrečany village (SC Slovakia). Molecular identification confirmed that these positive pools contained of two ornithophilic species; *Cx. pipiens pipiens* and *Cx. torrentium*. However according recent studies, host seeking activity in the *Cx. pipiens* complex is not as strict as was previously assumed and all member of the complex may serve as bridge vectors for arboviruses [34]. Given the high prevalence in vectors and widespread circulation of USUV, its risk to public health in Slovakia should be reconsidered.

The entomological surveillance conducted in this study provides some preliminary indications on the seasonal prevalence of WNV and USUV in Slovakia. All USUV infected mosquitoes were trapped between the months of June and October. The first human WNF case occurred in August 2019, the same month as all WNV infected mosquitoes identified in this study were trapped in 2018. In the previous study conducted in Slovakia, WNV and USUV infection of mosquitoes was observed between July and September [15]. According to present data, the seasonal prevalence of USUV appears to be longer than WNV, but the peak incidence for both viruses in vector mosquitoes seems to be in August, as suggested by the abundance of positive mosquito pools in that month. This finding may help healthcare practitioners and treating physicians to better detect and diagnose WNF and WNND cases, who may present in a very similar manner to other diseases, e.g. tick-borne encephalitis. The data will also further inform public health and veterinary authorities, allowing better seasonal targeting of prophylaxes and prevention activities in affected areas to protect human and animal health.

Some limitations of our study should be noted. No comprehensive WNV and/or vector control measures are established in Slovakia, so the selection of sampling areas was challenging. Sampling sites also changed between the 2 years of the surveillance, which could have resulted in differences in the 2018 and 2019 trapping seasons. Also, trap types varied according to the mosquito habitat as well as in their way of use. EVS traps were employed mainly in marshes and fishponds and BG-traps in the more urbanised areas. Nevertheless, the principle of both traps is the same – they attract female mosquitoes via carbon dioxide – and the results are likely comparable. Despite these limitations, our study adds to the overview of USUV and WNV ecology in Europe.

### Conclusion

We report the first human case of WNF from Slovakia and the results of a concurrent surveillance study undertaken to identify WNV and USUV foci, vectors, seasonal activities and the type of habitat where these viruses occur in Slovakia. The mosquito sampling sites were selected to cover three territorial units of southern Slovakia, where the circulation of these two arboviruses could occur, and one unit in the north east with a site at higher altitude. USUV was found at all investigated southern locations, while WNV was detected in the SC territorial unit as well as in the SW one, where the human WNF case occurred. The data obtained contribute to a preliminarily assessment of the public health risk of WNV and USUV in Slovakia. Continued mosquito surveillance will allow monitoring of these viruses’ activity in the country and help to preempt future human cases and outbreaks.
